# Increasing incidence and spatial hotspots of hospitalized endometriosis in France from 2011 to 2017

**DOI:** 10.1038/s41598-022-11017-x

**Published:** 2022-04-28

**Authors:** Joëlle Le Moal, Sarah Goria, Julie Chesneau, Arnaud Fauconnier, Marina Kvaskoff, Perrine De Crouy-Chanel, Vanessa Kahn, Emile Daraï, Michel Canis

**Affiliations:** 1 DATA Science Department, Santé publique France, 12 rue du Val D’Osne, 94415 Saint Maurice, France; 2 Gynaecology and Obstetrics CHI Poissy/Saint-Germain-en-Laye, Poissy, France; 3 Paris-Saclay University (UVSQ), Montigny-le-Bretonneux, France; 4 Paris-Saclay University, UVSQ, Univ. Paris-Sud, Inserm, Gustave Roussy, “Exposome and Heredity” Team, CESP, 94805 Villejuif, France; 5 Gynaecology and Obstetrics, Private practitionner, Paris, France; 6 Department of Obstetrics Gynaecology and Reproductive Medicine, Tenon Hospital, Paris, France; 7 Department of Obstetrics Gynecology and Reproductive Medicine, CHU Clermont Ferrand, Clermont Ferrand, France

**Keywords:** Endocrine reproductive disorders, Risk factors, Ecological epidemiology, Environmental impact

## Abstract

Endometriosis is a female hormone-dependent disease, possibly related to endocrine disruptor exposure. We aimed to monitor this disease nationwide in France and analyze spatial trends at a fine scale to explore possible environmental contributing risk factors. We conducted a retrospective national descriptive study from 2011 to 2017 in females aged 10 years old and over, using comprehensive hospital discharge data. Cases were identified using ICD-10 N80 codes and were localized at their municipality of residence. We defined incident cases as the first hospital stay of patients, without a stay in at least the previous 5 years. We performed statistical analyses according to age and type of endometriosis, and we modeled the temporal, spatial and spatiotemporal trends. We identified 207,462 incident cases of all-type hospitalized endometriosis (83,112 for non-adenomyosis cases). The crude incidence rate for the study period was 9.85/10,000 person-years (3.95/10,000 for non-adenomyosis cases). From 2011 to 2017, the risk of all-type endometriosis increased by 8.5% (95% CI: 3.9; 13.4) (by 3.6% (95% CI: 0.6; 6.8) for non-adenomyosis cases). The risk was geographically heterogeneous, with 20 high-risk hotspots, showing similar results for non-adenomyosis cases. Shifting practice patterns, improved awareness and healthcare disparities interlinked with environmental risk factors could explain these trends.

## Introduction

Endometriosis is a growing public health issue because of the high frequency suspected in the general female population^1,2^ and its heavy health consequences, especially in terms of chronic pelvic pain and infertility. However, fine and geographical epidemiologic data are lacking, especially in France, and its etiology is largely unknown. Defined as the growth of endometrial-like tissue outside of the uterine cavity, it is an inflammatory and hormone-dependent disease, exacerbated by estrogens, dampened by androgens or during menopause. Hence, etiologic hypotheses involve the role of endocrine disrupting chemical (EDC) exposure, with a growing body of evidence^3,4^. Several mechanisms of action could target the hormonal, immune, and inflammatory systems^5^.

Santé publique France, the French National Public Health Agency, has developed an epidemiological monitoring program to study reproductive health indicators relating to EDC exposure nationwide using existing databases. Based on the weight of evidence regarding its possible links to EDC exposure, we selected endometriosis as one of the key health concerns to monitor^6^. The main EDCs suspected to be related to endometriosis are bisphenol A, phthalates, dioxins, polychlorobiphenyls (PCBs), and organochlorine pesticides^7^. New evidence has recently been published regarding the latter three^3,4,8^ which are persistent chemical families in the environment and could be released through industrial or agricultural activities. In line with this hypothesis, a recent study has suggested a higher incidence of endometriosis in a heavily industrialized area in Italy^9^.

In this study, we aimed to describe for the first time the national incidence of hospitalized endometriosis in France for monitoring purposes, and to analyze the time and spatial trends at a fine scale to explore the possibility of local environmental contributing risk factors.

## Materials and methods

### Health and population data

In a previous feasibility study^10^ we built epidemiologic indicators to reflect incident cases of hospitalized endometriosis using the French national hospital discharge database included in the National Health Data System. This comprehensive database covers the whole of France since 2002 and includes discharges from both public and private hospitals^11,12^.

Two indicators were considered. For the first and main one, cases were identified using an algorithm that selects their surgical stay according to the diagnosis codes from the 10th edition of the International Classification of Diseases (ICD-10). The selected cases had a main, related, or associated ICD code for endometriosis (N800 to N809) to denote all types of hospitalized cases of endometriosis. We chose this main indicator because it was the most sensible to reflect the public health problem of endometriosis. The term “hospitalized cases of endometriosis” was chosen as it includes both patients requiring a diagnostic or operative laparoscopy for endometriosis but also patients with proven endometriosis on imaging techniques requiring medical treatment for severe pain as well as patients treated by ovarian punction under sonographic guidance for large symptomatic endometrioma.

Two subtypes of this main indicator were also considered: cases coded “isolated N800”, mainly composed of adenomyosis cases, which we named “adenomyosis cases,” and cases coded “everything except isolated N800”, which we named “non-adenomyosis cases.” However, it is important to precise that cases coded “isolated N800” may reflect several chirurgical forms of endometriosis of the uterus, with possible different models of pathogenesis. As non-adenomyosis cases are reputed to be more hormone-dependent than adenomyosis cases, these cases were particularly interesting for our environmental purposes, even though, in a recent review, both types could be related to endocrine disruptor exposure^13^.

In addition, we considered a secondary indicator, that was built differently, and which was more specific, in order to test the robustness of the results. The algorithm was defined using both specific ICD-codes and the French Common Classification of Medical Procedures (CCMP). It included 7 specific types of endometriosis which we considered homogeneously treated and coded around France: endometrioma, superficial endometriosis, deep endometriosis of the rectovaginal septum, intestinal endometriosis, ureteral endometriosis, vesical endometriosis, and parietal endometriosis. For example, to be identified as a case of endometrioma, patients had a main, related, or associated ICD code for ovary endometriosis (N801), and had in addition a CCMP code for unilateral ovariectomy, or for ovarian cystectomy, or for partial resection of the ovary etc. All these codes have been selected by co-authors surgeons to characterize the way endometrioma and the others specific types are usually coded in hospital. All algorithms are detailed in a previous publication^10^.

For each indicator, we defined incident cases as the first stay of patients for a diagnosis of endometriosis for the period 2011–2017, without a stay for such a diagnosis in at least the previous 5 years, in females aged 10 years and over. The study covered the whole of France, including its overseas territories. Patients were localized by their department of residence at the time of hospitalization. In France there are 100 departments. In Metropolitan France, patients were also localized using the municipality code of their place of residence at the time of hospitalization. In Metropolitan France, there are 35,798 municipality codes.

We extracted data according to the authorization of the National Commission for Data Protection and Privacy (CNIL) obtained by Santé publique France (N°902167).

The French National Institute for Statistical and Economic Studies (INSEE) provided population data by year, age, municipality, and department.

All methods were carried out in accordance with relevant guidelines and regulations in France.

### Descriptive analyses

We estimated the national incidence rate and the incidence rate according to age using the main indicator. We estimated the incidence rates according to year and subtypes of endometriosis.

### Spatiotemporal model at the department scale throughout France

We used the spatiotemporal model proposed by Knorr-Held^14–16^, an extension of the Besag, York, and Mollié (BYM) spatial model^17^. It is a Bayesian hierarchical model. The model decomposes disease risk into marginal spatial and temporal components, and a space–time interaction term. The interactions between space and time explain differences in the time trend of disease for different areas. Structured and unstructured random effects of time were included in the model. A second-order random walk was used to model the temporal trend. The BYM model was used to model spatially structured and unstructured random effects. Adjacency was used to define neighbors: departments sharing a border with the department of interest were defined as neighbors.

We modeled the number of cases with a Poisson distribution using a log-linear model. The model included age, modeled by a second-order random walk, and the population of females according to age, year, and department as an offset term.

Model fit was measured by the deviance information criterion and the effective number of parameters^18^.

### Spatial model at the municipality scale in Metropolitan France

A Poisson log-linear model was defined to estimate the spatial trend of the main indicator at the municipality level. Again, the Besag–York–Mollié model was used to model spatially structured and unstructured random effects. For comparative purposes, we also considered the model proposed by Leroux et al. ^19^ and the modified BYM proposed by Riebler et al. (^20^). Adjacency was used to define neighbors. The population of females according to age and municipality was included in the model as an offset term. The residuals from the BYM model were tested for spatial autocorrelation using a permutation test based on Moran’s I statistic (based on 10,000 random permutations)^21^.

The integrated nested Laplace approximation (INLA) approach was used to compute the posterior margins of all the parameters of interest^22^.

These models were implemented in the R software environment using the *mgcv*^23^, *INLA*^22^, and *CARBayes*^24^ packages.

We present the results as the percentage increase in the risk of endometriosis during the study period and its 95% credible interval, as well as municipality-specific relative risks (RRs) and their posterior probability to be greater than 1.

### Additional analyses

We carried out several additional analyses to inform the discussion. We estimated the incidence rates according to year and subtypes for the secondary indicator.

To argue for the possible role played by an increased use of pelvic magnetic resonance imaging (MRI) in the observed temporal trend, we analyzed the cases that underwent this examination 6 months before or 3 months after hospitalization. In terms of the spatial trends, we included in the model the incidence rate of non-endometriotic ovarian cysts, a disease that is different from endometriosis but treated by the same specialists.

Finally, to better define the areas at high risk of endometriosis, we conducted exploratory cluster detection in Metropolitan France at the scale of municipalities in women aged 25–49 years. Kulldorff’s spatial scan statistic was used^25,26^. In our study, the scanning window is represented by grouping neighboring municipalities within a maximum radius of 15 km. We adjusted for the population density and socio-economic level of municipalities using the French Deprivation Index (FDep)^27^. This detection study was run in SaTScan version 9.7^28^.

## Results

### Descriptive results

With the main indicator, in the whole of France for the period 2011–2017, there were 207,462 incident cases of hospitalized endometriosis in females aged 10 years and over. The crude incidence rate was 9.85/10,000 person-years (PYs) for the study period and 12.9/10,000 PYs in females aged 10–49 years.

Table 1 shows the steady upward trend of the incidence rate with the main indicator for the study period, whereas it is not obvious for non-adenomyosis cases, which accounted for 41% of all cases.

**Table 1:** Number of incident cases of the main indicator (all-type hospitalized cases coded endometriosis) and the sub-type “non-adenomyosis cases” and their crude incident rate for the study period in the whole of France, in females aged 10 years and above.

Year	Number of incident all-type cases coded endometriosis (incidence rate/10,000 person-years)	Number of incident non-adenomyosis cases (incidence rate/10,000 person-years)
2011	28,377 (9.58)	11,752 (3.97)
2012	29,304 (9.84)	11,978 (4.02)
2013	28,883 (9.65)	11,628 (3.88)
2014	29,801 (9.90)	11,849 (3.93)
2015	29,459 (9.74)	11,724 (3.88)
2016	30,401 (10.01)	12,042 (3.96)
2017	31,237 (10.24)	12,139 (3.98)
Total	207,462 (9.65)	83,112 (3.95)

Women aged 25–49 years represented the majority of all-type cases (68.3%), with an incidence rate of almost 19/10,000 PYs (Table 2). Females under 25 years of age accounted for less than 4% of the all-type cases and those aged 50 years and over accounted for 27.8% of cases. For non-adenomyosis cases, females under 25 years of age accounted for 8.5%, as those aged 50 years and over.

**Table 2:** Number of incident all-type hospitalized cases coded endometriosis and of non-adenomyosis cases, and their incidence rate by year according to age.

Age	Number of incident all-type cases (%)	Crude incidence rate/10,000 person-years	Number of incident “non adenomyosis” cases (%)	Crude incidence rate/10,000 person-years
< 25 years	8162 (3.9%)	1.99	7039 (8.5%)	1.71
25–49 years	141,791 (68.3%)	18.88	69,188 (83.2%)	9.21
> = 50 years	57,509 (27.8%)	6.09	6885 (8.3%)	0.71
Total	207,462	9.85	83,112	3.95

**Figure 1:**
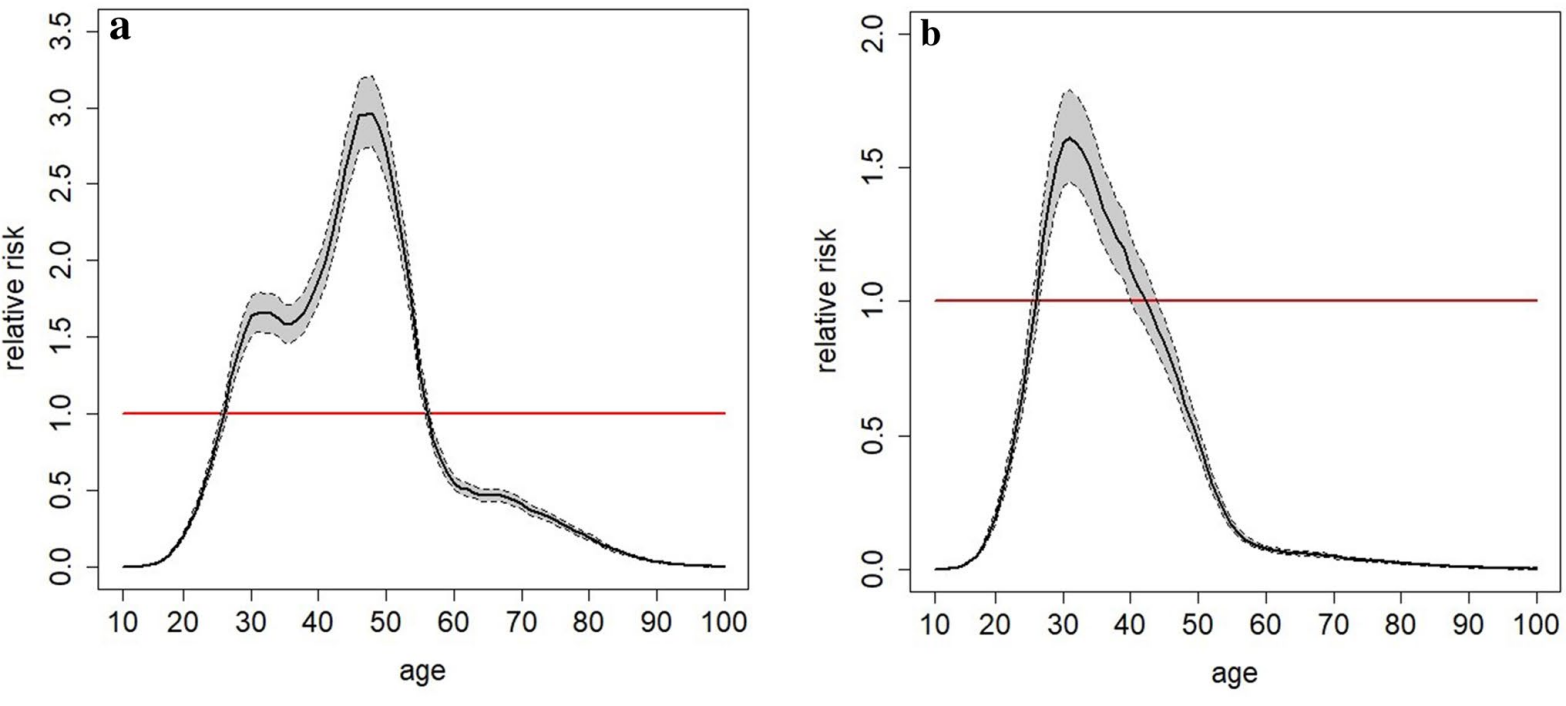
Relative risk of age, with 95% confidence interval compared to a reference case at 25 years (red line), (**a**) as regard all-type endometriosis cases, and (**b**) as regards non-adenomyosis cases.

Figure 1 presents the RR (and its 95% CI) of age compared to the reference at 25 years for all-type and non-adenomyosis cases. A pronounced increase in RR was observed, reaching a maximum value at around 47 years for all-type cases. The RR of being hospitalized for endometriosis at 47 years old was 2.97 (2.74, 3.21), more than at 25 years. Beyond that age, a decrease was observed. For non-adenomyosis cases a maximum value was observed at around 30 years with a decrease observed beyond that age. In Metropolitan France, the regions with the highest incidence rate for all-type cases in females aged 10 years and over were in the south-west and south: Nouvelle-Aquitaine (11.61/10,000 PYs) and Provence-Alpes-Côte d’Azur (10.98/10,000 PYs). In the overseas departments (Guadeloupe, Martinique, French Guyana, and Reunion Island), 7064 cases were observed, with the incidence rate being particularly high in the Reunion Island (15.18/10,000 PYs) and Martinique (12.96/10,000 PYs).

### Modeled temporal and spatiotemporal trends at the department level

**Figure 2:**
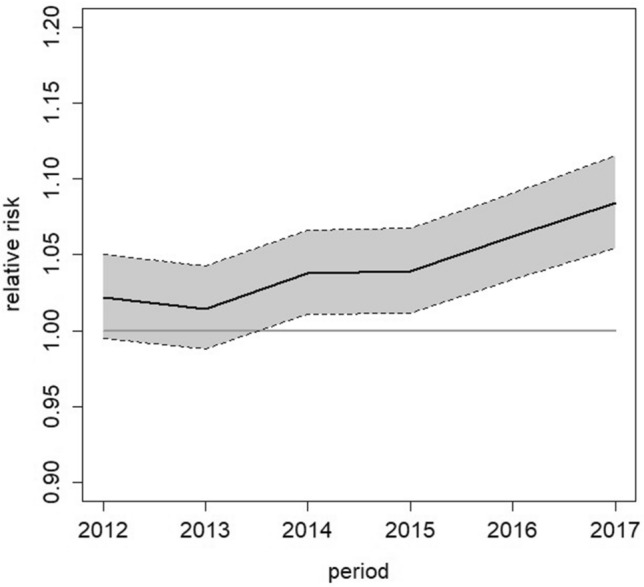
Temporal trend of the risk of all-type hospitalized cases in the whole of France in females aged 10 years and above: relative risk and its 95% confidence interval (Knorr-Held model).

In females aged 10 years and over, we observed an increase in the risk of hospitalized endometriosis for all-type cases over the study period. Figure 2 shows the estimated RRs for each year of the study period compared to 2011. The overall estimated increase was equal to 8.5% (95% CI: 3.9; 13.4).

The risks for “adenomyosis cases” and “non-adenomyosis cases” increased by 15% (95% CI: 9.9; 20.4) and 3.6% (95% CI: 0.6; 6.8), respectively, over the study period. In women aged 25–49 years, the risk for all-type cases increased by 10.4% (95% CI: 6.2; 14.8), while no increase was observed in in females under 25 years of age (Table 3).

**Table 3:** Temporal trend of the risk of all-type hospitalized cases coded endometriosis according to age.

Age	Relative risk	95% confidence interval
10–24 years	0.997	(0.921; 1.079)
25–49 years	1.104	(1.062; 1.148)
> = 50 years	1.031	(0.973; 1.093)

**Figure 3:**
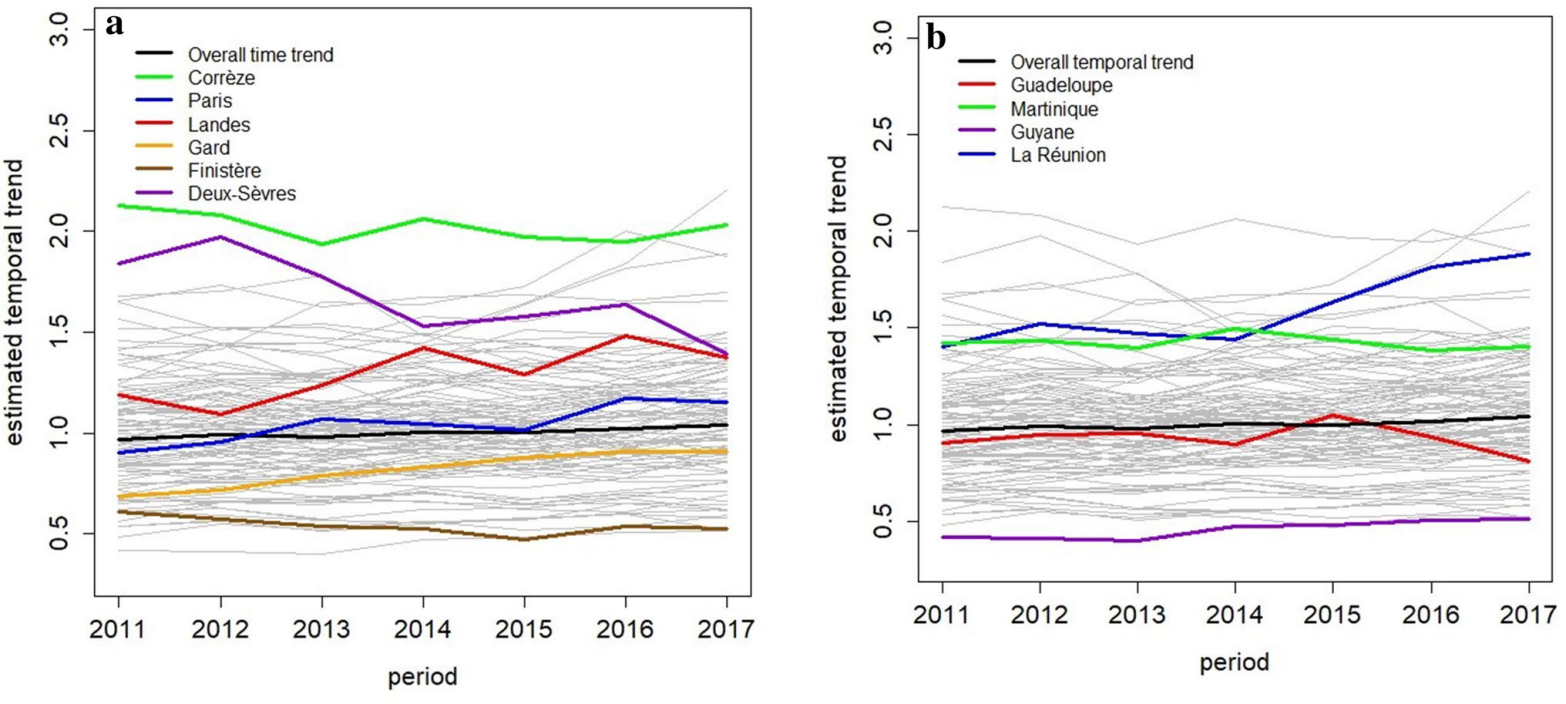
Estimated temporal trends (colored) for all-type cases compared to the national trend (black) (**a**) in some departments of metropolitan France and (**b**) in overseas departments.

We observed a spatiotemporal interaction for all-type hospitalized cases. Indeed, the temporal trends of several departments evolved differently than the national temporal trend. Figure 3 shows the estimated temporal trends of departments and highlights those with a sharper increase in the risk of hospitalized endometriosis compared to the national level [e.g., Landes and Gard in Metropolitan France (Fig. 3a)] and those with a declining trend (e.g., Finistère and Deux-Sèvres) during the study period. In Corrèze, the risk was quite steady but noticeably higher than the national trend throughout the study period. In the overseas departments (Fig. 3b), the Reunion Island showed a sharper increase in risk compared with the national level.

A spatial heterogeneity was observed with several departments showing a higher RR (Supplementary Fig. S1).

### Modeled spatial trends at the municipal level

**Figure 4:**
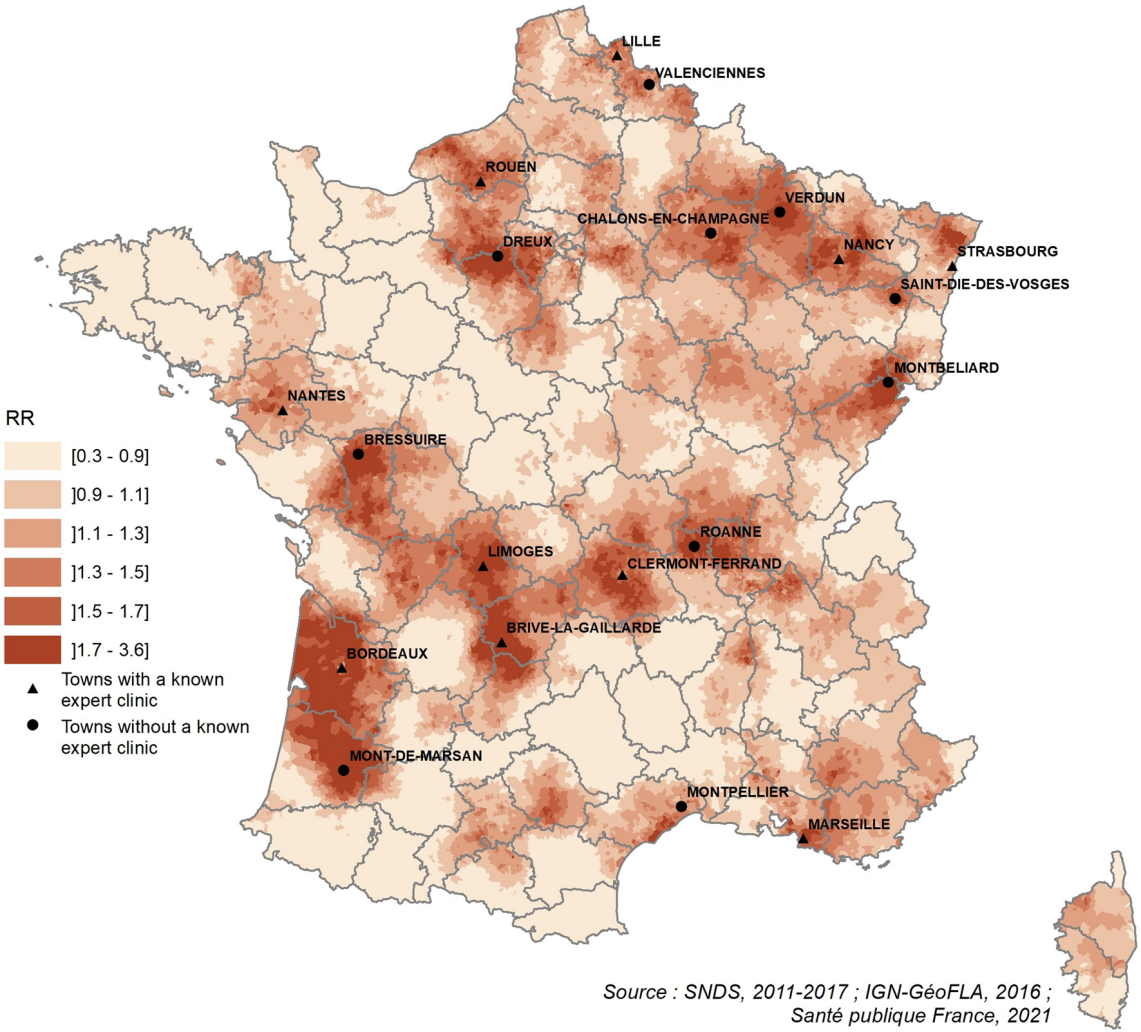
Relative risks (RR) of all-type hospitalized cases coded endometriosis in females aged 10 years and above at the municipal scale.

We also observed a heterogeneous spatial distribution of the risk of endometriosis at the municipality level for all-type cases. Around 20 areas scattered throughout France showed a high RR compared to the national risk. Among these 20 hotspots, 10 were located close to known expert clinics (Fig. 4). For non-adenomyosis cases, the picture was very similar (Supplementary Fig. S2).

## Discussion

This first national descriptive study used an indicator, which comprehensively reflects incident all-type hospitalized cases coded endometriosis in the French territory up to the municipality scale. We observed an increase in the risk of being hospitalized from 2011 to 2017 and spatial heterogeneity with the identification of 20 scattered hotspots in Metropolitan France as well as in 2 overseas departments.

### Descriptive results

The annual incidence rate (12.9/10,000 PYs) of all-type hospitalized cases coded endometriosis in France in females aged 10–49 years was of the same order of magnitude as the rates observed in other countries (Italy, Iceland) using similar methods^29,30^. Moreover, a recent meta-analysis^2^ estimated the pooled incidence rate of endometriosis based on hospital data to be 13.6/10,000 PYs (95% CI: 10.9; 16.3), which situates the French estimation within the confidence interval and close to the pooled value.

In our study, 68.3% of all-type cases and 83.2% of non-adenomyosis cases were aged 25–49 years, and only 3.6% (8.5% for non-adenomyosis cases) were under 24 years. In young females, this low percentage could reflect underdiagnosis or delayed diagnosis, because histologic evidence may occur after an interval of 5–10 years following the first signs of endometriosis^31^. Moreover, many cases are fortuitously diagnosed during fertility check-ups, which rarely take place before 25 years of age. This age distribution in France is close to the distribution observed in a recent Italian study (3.6% < 25 years, 76.4% in 25–49 years, and 21% > 50 years) carried out using similar methods in the population of the Friuli Venezia Giulia region from 2011 to 2013^30^. The Italian authors remarked a noticeable percentage of incident cases over 50 years of age for non-adenomyosis cases (11.5%), close to our results (8.3%), even though endometriosis is expected to attenuate after menopause. They suggested that endometriosis deposits could still be potentially active in older patients and be reactivated in the presence of certain hormones^30^. This hypothesis seems quite relevant regarding the potential link with EDC exposure. Indeed, the developmental hypothesis supposes that reproductive disorders at adult age could result from early (i.e., prenatal, perinatal, or pubertal) exposure to EDCs in specific exposure windows. In males, this hypothesis has been especially developed according to the so-called “testicular dysgenesis syndrome (TDS)”^32^. The disruption of fetal androgen action with EDCs, specifically in the “masculinization programming window” (MPW), induces a shorter anogenital distance that is supposed to provide a life-long readout of the level of androgen exposure in the MPW^33^ and is consistently associated in animals and humans with TDS troubles (cryptorchidism, hypospadias, poor sperm quality)^34^.

In females, the mirror concept of “ovarian dysgenesis syndrome” has been proposed, including a higher risk to develop endometriosis^35^. Interestingly, endometriosis has recently been associated with a shorter anogenital distance in women^36^, and this anthropological indicator, measurable using MRI, could be useful for a non-invasive diagnosis of the disease^37^.

In addition, some authors suggest that endometriosis onset could occur in two steps: an early hormonal-developmental step and a second hormonal step at adult age^38,39^, or a first initiation step with a second promotion step based on experimental tumor production^40^. Overall, these hypotheses could contribute to the unexpected proportion of hospitalized endometriosis cases identified after menopause. Another explanation could be the large number of fortuitous diagnoses of endometriosis at the same time as hysterectomies performed for diverse indications in women at an older age.

### Temporal trends

Studies on the temporal trends of endometriosis incidence used diverse methods and delivered differing results according to the country as reviewed in a recent study^1^. Only three studies carried out with hospital data in the general population are available. A Finnish study showed a decrease in incidence from 1987 to 2012^41^. An Icelandic study did not conclude to any trend from 1981 to 2000^29^, and a recent Korean study only showed an incidence increase in young women aged 15–19 and 20–24 years, but not in other age groups^42^.

In France, the increase in the risk of being hospitalized, observed for both adenomyosis and non-adenomyosis cases, could reflect a real increase in the incidence of endometriosis, consistent with the perception of numerous clinicians. We did not observe an upward trend in females under the age of 25 years, which could reflect the underdiagnosis of this population. The global increase could also relate to the increasing use of non-invasive examinations, like ultrasounds or pelvic MRI during the study period. Pelvic MRI was only recommended by the French Health Authority at the end of the study period^43^, although clinicians would have anticipated this recommendation, which is supported by the results of the additional analyses (Supplementary Material). In the study period, there was a 69% increase in cases who underwent this examination concurrently with hospitalization, which accounted for around a third of cases. The increasing use of MRI (or ultrasounds) would result in more and more cases treated without hospitalization and could explain the apparent increase of hospitalized incidence at later ages and less at younger ages.

Regarding the secondary indicator, the incidence rate in the whole of France during the study period remained steady. However, the trends differed according to each type (Table 4). The risk did not increase for endometrioma, a type of endometriosis that is not expected to depend on the use of pelvic MRI, but it did increase for intestinal endometriosis, expected to be strongly influenced by pelvic MRI. Therefore, these results also support the role of pelvic MRI. As for the divergent evolution of specific types of endometriosis, experts believe that it could depend on shifting practice patterns such as the more frequent tendency to medically treat endometrioma.

**Table 4:** Number of incident cases of hospitalized endometriosis and crude incident rate for specific types of endometriosis for the study period in the whole of France, in females aged 10 years and above.

Years	Endometrioma		Superficial endometriosis		Deep endometriosis of the rectovaginal septum		Intestinal endometriosis		Ureteral endometriosis		Vesical endometriosis		Parietal endometriosis	
	Number of cases	Crude incidence/10,000 PY	Number of cases	Crude incidence/10,000 PY	Number of cases	Crude incidence/ 10,000 PY	Number of cases	Crude incidence/10,000 PY	Number of cases	Crude incidence/10,000 PY	Number of cases	Crude incidence/10,000 PY	Number of cases	Crude incidence/10,000 PY
2011	3831	1.29	3847	1.30	1046	0.35	296	0.10	17	0.006	65	0.022	107	0.036
2012	3844	1.29	3945	1.32	1179	0.40	349	0.12	17	0.006	87	0.029	118	0.040
2013	3724	1.24	3786	1.26	1278	0.43	382	0.13	18	0.006	80	0.027	109	0.036
2014	3508	1.17	3940	1.31	1464	0.49	527	0.18	19	0.006	73	0.024	132	0.044
2015	3419	1.13	3755	1.24	1430	0.47	600	0.20	33	0.011	74	0.024	108	0.036
2016	3264	1.07	3835	1.26	1658	0.55	682	0.22	21	0.007	116	0.038	150	0.049
2017	3075	1.01	3999	1.31	1733	0.57	708	0.23	37	0.012	84	0.028	113	0.037
Total	24,665		27,107		9788		3544		162		569		837	
%	37		40.6		14.7		5.3		0.2		0.8		1.2	

*PY* person-year.

Another factor could also contribute to the global increase in hospitalized endometriosis. Several patient societies (EndoFrance, Endomind, Info-endometriose) have strongly advocated for better detection and care of this disease and provided targeted information, which may have resulted in increased awareness of patients and clinicians regarding the disease during the study period.

These factors are likely interlinked with a possible real increase in endometriosis incidence, which could be confirmed by a longer monitoring period.

### Spatiotemporal and spatial trends

The spatiotemporal and spatial heterogeneity of the risk of hospitalized endometriosis that we observed in France during the study period could be related to spatial disparities and different evolutions in terms of detection and hospital care. In half of the 20 hotspots in Metropolitan France, we identified a town where an expert clinic for endometriosis was operational during the study period (Fig. 4). In the overseas departments, we identified an expert clinic in the Reunion Island, where we also observed a high incidence. However, we identified expert clinics in areas with a low or moderate risk of hospitalized endometriosis, especially in Paris (four expert clinics), Lyon (two expert clinics), Rennes, Brest, and Angers. Adjusting the spatial model at the department scale with the density of gynecologists and obstetricians using the available data provided by the shared inventory of health professionals from 2011 to 2016 did not change the geographic distribution (data not shown). Adjusting for incident cases of non-endometriotic ovarian cysts only brought about some changes in several departments in the north where the risk attenuated, even though it stayed above 1 (data not shown).

Taken together, these results indicate that the activity of local expert clinics could only partially explain the spatial and spatiotemporal heterogeneity of the risk of hospitalized endometriosis. The contribution of environmental factors remains possible and plausible, as we argued above.

The results of the exploratory cluster detection performed in Metropolitan France showed a negative relation with the socioeconomic deprivation index. Indeed, a high socioeconomic status (SES) or education level has been associated with a higher frequency of endometriosis^44,45^, which probably reflects the better detection and patient care of women with high SES. However, this relation was inverted in a recent Swedish study, although the authors partly attribute this inconsistent finding to egalitarian health care in Sweden^46^.

Among the 40 detected clusters (p < 0.0001) in Metropolitan France, 23 were located in cities or their outskirts where expert clinics have been identified. Hence, even if there were some clues about the high industrial or agricultural activity in several cluster areas, this exploratory analysis did not allow us to further develop the environmental hypotheses.

### Limitations

The major limitation of the study is the potential variability of the results due to the local clinical practices and their evolution over time, which complicates the interpretation of our results, especially in the evolving field of endometriosis. We tried to minimize this bias by carrying out additional analyses. In addition, the indicator that we used only targeted the hospitalized cases of endometriosis, and therefore we underestimated the incidence of the disease. In the literature, the incidence estimated by cohorts in the general population is about three times the incidence of hospitalized endometriosis^2^. We know that coding errors may also occur, although in a monitoring system, this should not bias the results if the errors are steady in time and homogeneous across the territory. Finally, we did not have individual information about specific factors such as BMI, alcohol or tobacco consumption, nutrition, or lifestyle, which could have been important to further develop the analyses. Nevertheless, the indicator used here allows for the comprehensive and long-term monitoring of the whole territory, which was our purpose.

## Conclusions

These results provide for the first time a detailed overview of the epidemiology of hospitalized endometriosis in France, and they will be useful to estimate the public health burden of the disease. The increasing temporal trend could reflect a real increase in hospitalized endometriosis incidence, interlinked with the increased use of non-invasive diagnoses like ultrasounds and pelvic MRI and enhanced awareness of patients and clinicians about the disease. Nevertheless, a longer monitoring period will be necessary to confirm this trend. The observed spatial heterogeneity provided some possible clues consistent with the EDC hypothesis, although the heterogeneity of the healthcare offer for endometriosis seemed to have at least partially influenced the results. This work is the first step of long-term monitoring of endometriosis in France, and it will allow us to target future ecological studies to better explore environmental hypotheses.

## Supplementary Information

Supplementary Information.

## Data Availability

According to data protection and the French regulation, the authors cannot share the data used in this study. However, any person or structure, public or private, for-profit or non-profit, is able to access the National Health Data System (SNDS, https://www.snds.gouv.fr) upon authorization from the National Computers and Privacy Commission (CNIL), in order to carry out a study, a research or an evaluation of public interest.
