# Supplementary material for: Increasing incidence and spatial hotspots of hospitalized endometriosis in France from 2011 to 2017
Source: Sci Rep. 2022 Apr 28;12:6966. doi: 10.1038/s41598-022-11017-x (PMC9050825; doi:10.1038/s41598-022-11017-x)

Increasing incidence and spatial hotspots of hospitalized endometriosis in France from 2011 to 2017

Joëlle Le Moal

Sarah Goria

Julie Chesneau,

Arnaud Fauconnier,

Marina Kvaskoff

Perrine De Crouy-Chanel

Vanessa Kahn,

Emile Daraï

Michel Canis

Supplementary Material

To argue for the possible role of an increased use of pelvic magnetic resonance imaging (MRI) in the temporal trend, we analyzed the incident cases using this examination. As it is not possible to specifically identify “pelvic” MRI in the discharge database, we identified cases with the examination contemporary with hospitalization (6 months before or 3 months afterwards). In the discharge database, we only could identify cases with ambulatory examinations or external consultations in private hospitals.

We identified 109,601 reimbursements for MRI linked to the cases, accounting for 35% of total cases. Among them, 36,349 cases had the examination 6 months before or 3 months after hospitalization with the diagnosis of endometriosis, thus suggesting that it was a pelvic MRI.

Incident cases with hospitalized endometriosis with a probable pelvic magnetic resonance imaging scan according to year		
Year of incidence	Number	%
2011	3,825	10.52
2012	4,328	11.91
2013	4,692	12.91
2014	5,061	13.92
2015	5,594	15.39
2016	6,380	17.55
2017	6,469	17.80

The above table shows the evolution of the number and percentage of cases. There is a steady increase in the percentage of probable pelvic MRI linked to the incident cases of hospitalized endometriosis, with an increase of 69% of cases during the study period.

Supplementary Figure S1

Department-specific relative risks (RRs) and the probability that these RRs are greater than 1 for all-type hospitalized cases.

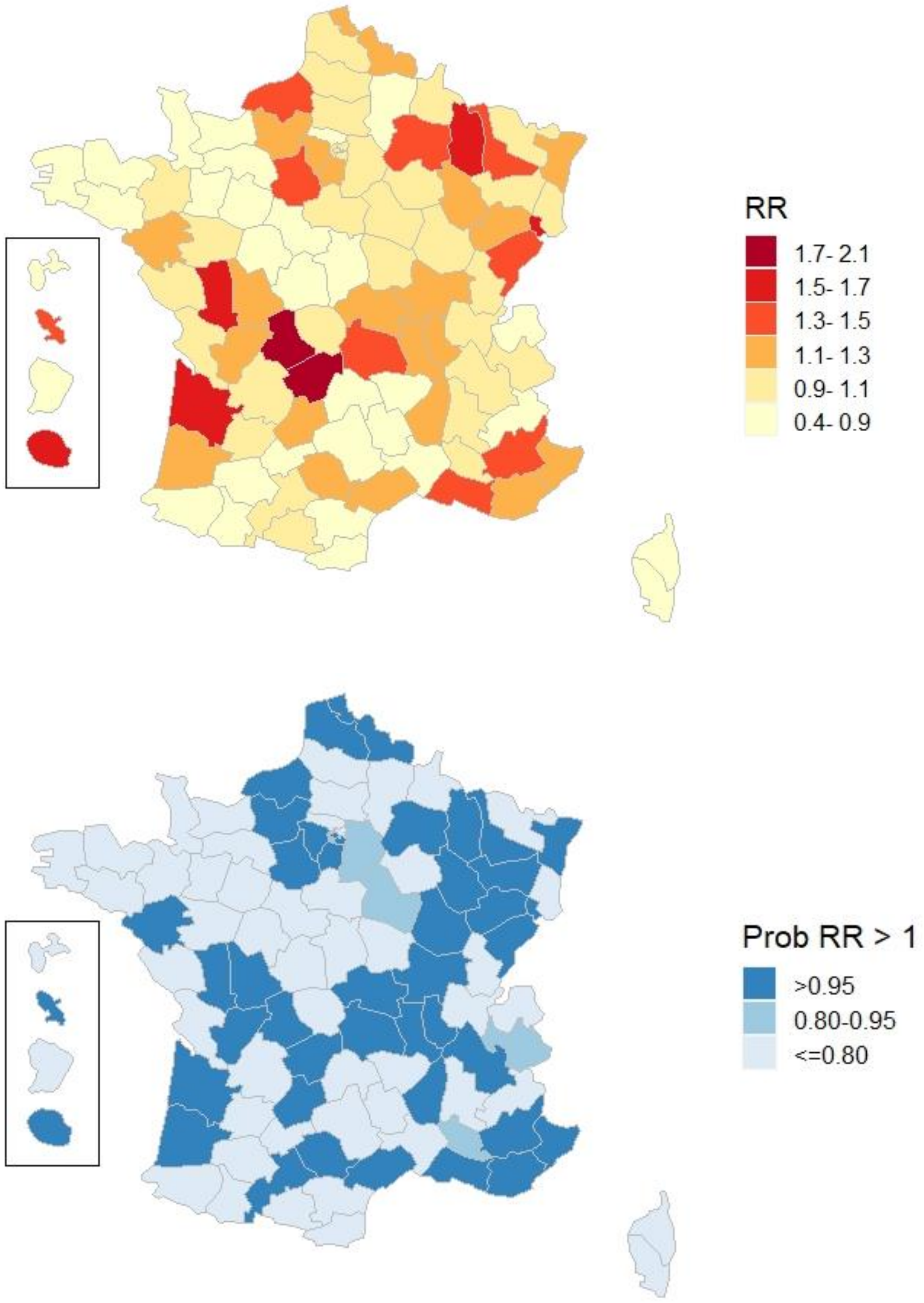

Supplementary Figure S2

Relative risks (RR) of non-adenomyosis cases in females aged 10 years and above at the municipal scale

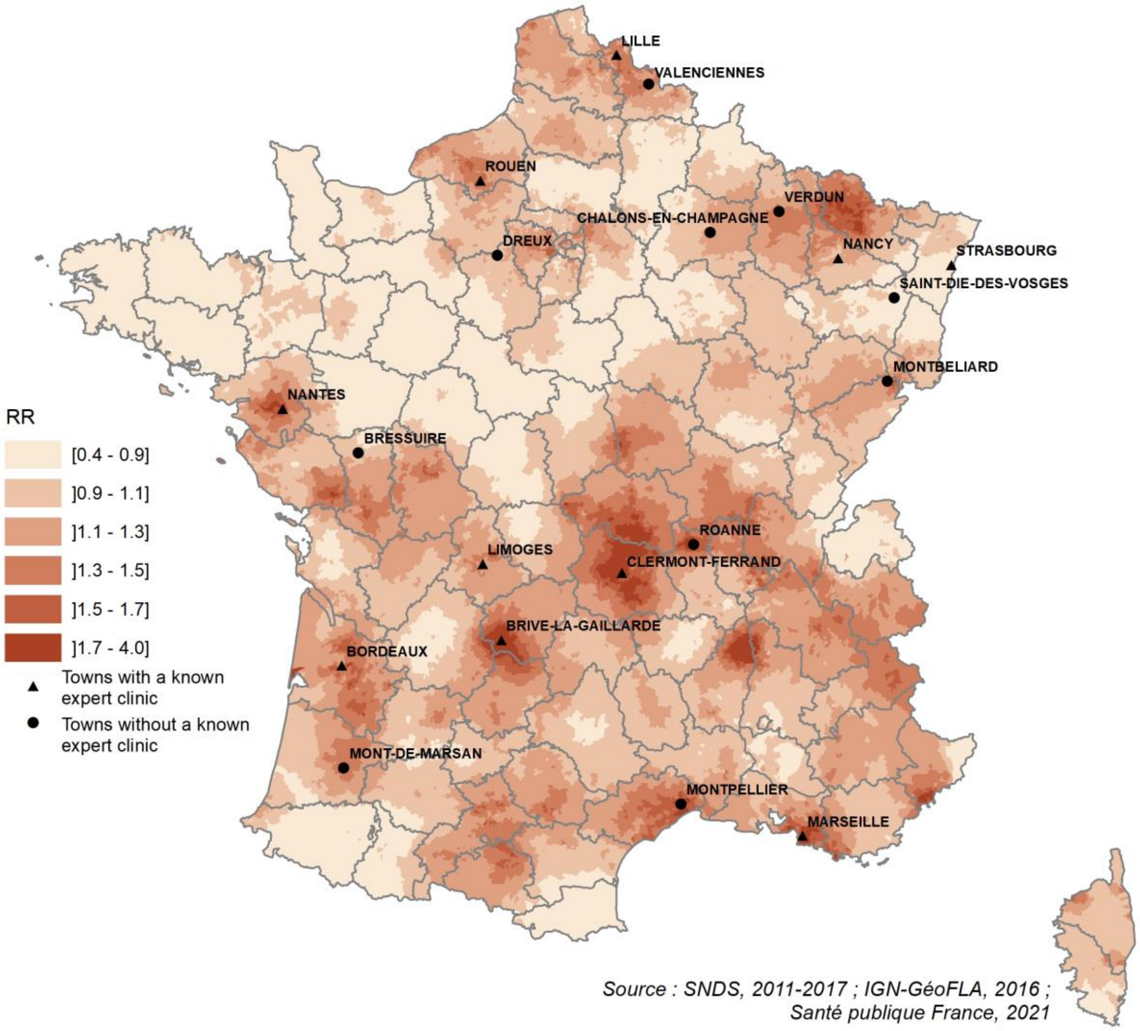

Supplement: Supplementary file 1 — Supplementary Information. [file 41598_2022_11017_MOESM1_ESM.pdf]
